# Cardiovascular Medicine Use Among Adults With ADHD: A Nationwide Study in Australia

**DOI:** 10.1177/10870547261418763

**Published:** 2026-03-27

**Authors:** Masako Araki, Helga Zoega, Malcolm Gillies, Michael O. Falster, David Peiris, Sallie-Anne Pearson, Henrik Larsson, Juliana de Oliveira Costa

**Affiliations:** 1 UNSW Sydney, Australia; 2 University of Iceland, Reykjavík, Iceland; 3 Örebro University, Sweden; 4 Karolinska Institutet, Stockholm, Sweden

**Keywords:** ADHD, adults, cardiovascular medicines, cardiovascular comorbidities, cardiovascular diseases

## Abstract

**Background:**

Despite cardiovascular conditions being common in adults with ADHD, data on patterns of cardiovascular medicine use in this population are scarce.

**Methods:**

Using dispensing claims for a 10% random sample of Australians, this population-based study comprised 14,753 adults with ADHD (defined as having ≥2 ADHD medicine dispensings in 2012–2020) who were 1:4 sex- and age-matched with 59,012 adults without ADHD (no ADHD dispensings). We estimated the prevalence of cardiovascular medicine use in 2021 among adults with and without ADHD, overall and by medicine type, sex, and age. Using Poisson regression, we calculated age- and sex-adjusted prevalence ratios (PRs) with 95% confidence intervals (CIs) to assess associations.

**Results:**

Overall cardiovascular medicine use was more prevalent among adults with ADHD than those without (16.5% vs. 10.0%, aPR = 1.7, 95% CI [1.6, 1.7]), with the highest difference among those aged 18 to 29 years (aPR = 2.8, 95% CI [2.4, 3.1]). We observed increased differences in use of propranolol (3.2% vs. 0.7%), loop diuretics (0.8% vs. 0.4%), potassium-sparing diuretics (0.9% vs. 0.4%), cardiac therapy (0.8% vs. 0.5%), and antithrombotic agents (2.2% vs. 1.4%). Among females, we noted associations of ADHD with specific diuretic subgroups (loop, aPR = 2.8, 95% CI [2.1, 3.7]; potassium-sparing, aPR = 2.5, 95% CI [1.9, 3.3]).

**Conclusion:**

We identified an elevated prevalence of cardiovascular medicine use among adults with ADHD, particularly among younger people. We also observed distinct patterns in specific medicine use between sexes, suggesting potential sex-specific effect modification. Our findings underscore the importance of regular monitoring and management of cardiovascular health among people with ADHD across the lifespan.

## Introduction

ADHD is a common neurodevelopmental condition characterised by impulsivity, hyperactivity, and inattention. The estimated prevalence is 5% to 7% in childhood and 3% in adults (Di Lorenzo et al., 2023; Posner et al., 2020; Thomas et al., 2015), with two-thirds of ADHD diagnosed in childhood persisting into adulthood (Simon et al., 2009).

Emerging evidence highlights an association between ADHD and cardiovascular disease (CVD; Li et al., 2022, 2023). The observed higher burden of CVD in people with ADHD might be due to poorer health behaviours (Treur et al., 2021), genetic predisposition for cardiometabolic conditions (Du Rietz et al., 2021; Garcia-Argibay et al., 2022), and psychiatric comorbidities (Choi et al., 2022; Zhang et al., 2022). At the population level, a recent longitudinal cohort study suggests that people with ADHD have a two-fold risk of developing cardiovascular conditions, even after accounting for associated risk factors (e.g., unhealthy lifestyle habits, alcohol use, drug use, and ADHD medicines; Li et al., 2022). Moreover, the rising number of adult ADHD diagnoses and the use of long-term ADHD medicines and concomitant psychotropic medicine use raise concerns about their potential effects on the cardiovascular system (e.g., hypertension; Zhang et al., 2022, 2024).

Current knowledge about treatment with cardiovascular medicine in people with ADHD is limited (Zhang et al., 2021); a better understanding of prescribing patterns is needed to guide screening and treatment options. People living with ADHD are likely to have sex-specific biological and behavioural factors (Vogel et al., 2021), that may result in distinct profiles of cardiovascular conditions and medicine use. Females with ADHD are underdiagnosed, and prone to comorbid psychiatric conditions that may be linked to an increased cardiovascular risk (Young et al., 2020). Additionally, CVD is influenced by lifelong risk factors (e.g. substance use and eating disorders; Du Rietz et al., 2021; Li et al., 2022, 2023) that are typically pronounced in people with ADHD from an early age. Furthermore, given the diverse spectrum of cardiovascular conditions, with varying severity, the types of medicines use in this population may also vary. Therefore, understanding sex- and age-specific real-world medicine utilisation of cardiovascular medicine types is critical for optimising pharmacotherapy and effectively managing people with ADHD and cardiovascular comorbidities.

Leveraging national dispensing claims for a 10% random sample of people, we aimed to describe and compare patterns of cardiovascular medicine use among adults with and without ADHD. We hypothesised that adults with ADHD would have a higher prevalence of cardiovascular medicine use than adults without ADHD because of differing cardiovascular risk profiles; and that age and sex might play a role in such differences.

## Methods

### Setting and Data Source

Australia maintains a universal healthcare system, providing citizens, permanent residents, and eligible visitors with access to publicly subsidised medicines through the Pharmaceutical Benefits Scheme (PBS). The scheme subsidises the cost of the medicine, while the recipient also contributes a fixed co-payment depending on their beneficiary status and the cost of medicine, ensuring accessibility and affordability of medicines (Mellish et al., 2015).

In this study, we used a 10% sample of the PBS-eligible population (PBS 10% sample). This is a random sample extracted based on each individual’s unique identifier. The PBS 10% sample, provided by Services Australia, contains individual-level data on PBS-listed medicines dispensed at community pharmacies, private hospitals, and on discharge from public hospitals (in most states), including information on medicines dispensed (PBS item code and dispensing date) and patient year of birth, sex, and year of death. However, it does not capture over-the-counter medicines, private prescriptions, or medicines dispensed to public hospital in-patients (Mellish et al., 2015). We used PBS item codes to derive the generic name and the World Health Organization Anatomical Therapeutic Chemical (ATC) code for each dispensed medicine (Mellish et al., 2015). To protect privacy, supply dates are randomly perturbed by ±14 days for each individual patient (Mellish et al., 2015).

### Study Design and Population

This was a cross-sectional study comparing cardiovascular medicine use in a single calendar year (2021) among adults with and without ADHD. We used PBS 10% data to identify “adults with ADHD,” defined as individuals aged 18 years and over as of January 1, 2021, who had received at least two dispensings of PBS-listed ADHD medicines during the period from July 1, 2012 through December 31, 2020 (Zhang et al., 2021). Given the stringent regulations governing psychostimulants in Australia where ADHD medicine prescription requires a formal diagnosis of ADHD by a specialist, the prescription can be used as a proxy for ADHD diagnosis (Australian ADHD Professionals Association, 2022; Bruno et al., 2022; Zhang et al., 2021). We identified ADHD medicines using the ATC codes (generic name [ATC code]: methylphenidate [N06BA04], dexamfetamine [N06BA02], lisdexamfetamine [N06BA12]), atomoxetine [N06BA09], and guanfacine [C02AC02]; Supplemental eTable S1).

As a comparison group, we defined “adults without ADHD” as those with no record of PBS dispensing of ADHD medicines during the period from July 1, 2012 through December 31, 2020. Therefore, people with only one dispensing of ADHD medicines were excluded from the study. We randomly matched “adults without ADHD” 1:4 on sex and year of birth with “adults with ADHD.”

All included adults had to be alive on December 31, 2021, that is, at the end of the study period. A diagram of study population selection and graphical representation of the study design are presented in the Supplemental Material (Supplemental eFigures S1 and S2).

### Cardiovascular Medicines of Interest

We identified cardiovascular medicines dispensed between January 1, 2021, and December 31, 2021. We ascertained use of cardiovascular medicines, based on the ATC classification (Supplemental eTable S2): antithrombotic agents (B01), cardiac therapy agents (C01), lipid modifiers (C10), and antihypertensives, including diuretics (C03), beta-blockers (C07), calcium-channel blockers (C08), renin-angiotensin-aldosterone system inhibitors (RAASi; C09), and other antihypertensives (C02). We counted each component of fixed-dose combinations separately under the ATC for the component; for example, records for the combination of atorvastatin and amlodipine were counted as C10AA05 and C08CA01 rather than C10BX03.

We further classified cardiovascular medicine types into the following subgroups: diuretics (low-ceiling, loop, and potassium-sparing), beta-blockers (nonselective and selective), calcium-channel blockers (dihydropyridine and non-dihydropyridine); and RASSi (angiotensin receptor blockers [ARB] and angiotensin-converting enzyme inhibitors [ACEI]; Poluzzi & Korhonen, 2016).

We did not assess the use of clonidine, prazosin, minoxidil, and epinephrine, as these medicines are more frequently prescribed for non-cardiovascular conditions (Butt et al., 2022). Nor did we assess aspirin use due to its over-the-counter availability and consequently under ascertainment in our dataset. We conducted a post hoc analysis excluding the use of propranolol, a medicine often prescribed for non-cardiovascular conditions as well as for CVDs (Lin et al., 2006; NHFA CSANZ Heart Failure Guidelines Working Group et al., 2018).

### Data Analysis

To describe the study population characteristics at baseline (January 1, 2021), we presented data on sex (male, female), age group (18–29, 30–49, 50–64, and ≥65 years), and ADHD medicine type as absolute frequencies and percentages. We also described the specific year and ADHD medicine used for the most recent ADHD dispensing before the baseline. Among adults identified as with and without ADHD, we described pre-existing conditions at baseline, based on dispensing claims from the previous year (2020) using the Rx-Risk-V Comorbidity Index algorithm (Pratt et al., 2018). Comorbidities of interest included alcohol dependence, anxiety, bipolar disorder, depression, migraine, pain, psychosis, smoking cessation, diabetes, arrhythmia, congestive heart failure, and ischaemic heart disease (Pratt et al., 2018).

We measured the frequency of adults receiving at least one cardiovascular medicine dispensing in 2021 and estimated the prevalence of cardiovascular medicine use as a percentage, by medicine type (i.e., medicine class, subgroup, and specific medicines), sex and age group. Additionally, among people with ADHD we presented the 20 most frequently used cardiovascular medicines, overall and stratified by sex. We then compared prevalence of cardiovascular medicine use between adults with ADHD and age- and sex-matched adults without ADHD, adjusting for age and sex, using Poisson regression. We expressed results as prevalence ratios (PRs) with 95% confidence intervals (CIs) based on robust standard errors.

All analyses were performed with SAS version 9.4 (SAS Institute), and figures were created using R Statistical Software (v4.1.1; R Core Team 2021). We reported our results following the RECORD-PE statement for pharmacoepidemiological studies (Supplemental eTable 3; Langan et al., 2018).

## Results

### Characteristics of Study Population

We identified 14,753 adults with ADHD, over 60% of whom were male. Around 40% were aged 18 to 29 years, over 40% were aged 30 to 49 years, about 13% aged 50 to 64 years and the remaining 3% aged ≥65 years. Based on the latest ADHD medicine dispensing at baseline, approximately 60% received their latest ADHD medicine within the year prior to the baseline, specifically in 2020. Adults with ADHD were more likely than those without ADHD to have been treated for psychiatric conditions (e.g., depression, anxiety, and psychosis) and physical conditions (e.g., pain, diabetes, and ischaemic heart disease; Table 1).

**Table 1.** Characteristics of Australian Adults With and Without ADHD Matched on Age and Sex.

Characteristic	Adults with ADHD		Adults without ADHD	
Total, *n* %	14,753	100%	59,012	100%
Age group				
18–29	6,116	41.5	24,464	41.5
30–49	6,349	43.0	25,396	43.0
50–64	1,839	12.5	7,356	12.5
≥65	449	3.0	1,796	3.0
Sex, *n* %				
Female	5,571	37.8	22,284	37.8
Male	9,182	62.2	36,728	62.2
Medicine use for comorbidities, based on Rx-Risk-V^a^,*, *n* %				
Alcohol dependence	103	0.7	78	0.1
Anxiety	2,035	13.8	1,625	2.8
Bipolar	264	1.8	102	0.2
Depression	5,137	34.8	5,613	9.5
Migraine	386	2.6	588	1.0
Pain	2,828	19.2	5,187	8.8
Psychosis	1,593	10.8	870	1.5
Smoking cessation	212	1.4	177	0.3
Diabetes	624	4.2	5,613	9.5
Arrhythmia	38	0.3	113	0.2
Congestive heart failure	46	0.3	78	0.1
Ischaemic heart disease (angina)	56	0.4	126	0.2
Ischaemic heart disease (hypertension)	1,080	7.3	2,042	3.5

a Rx-Risk: Rx-Risk Comorbidity Index.

* Probability values (*p* values) were calculated for the difference in proportions between adults with and without ADHD for each comorbidity with all the *p* values being less than .0001.

### Prevalence of Cardiovascular Medicine Use by Age and Sex

**Figure 1.**
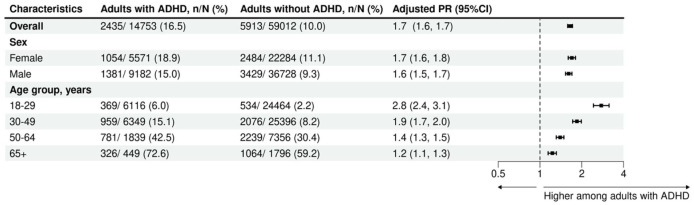
Prevalence of overall cardiovascular medicine use among Australian adults with and without ADHD, matched on age and sex. *Note*. PRs were adjusted for age and sex.

**Figure 2.**
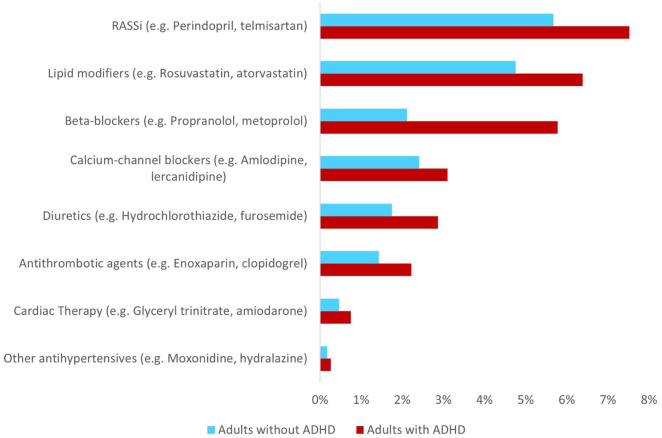
Prevalence of cardiovascular medicine use among Australian adults with and without ADHD, matched on age and sex-matched by cardiovascular medicine type. *Note*. Names in parentheses are the generic names of the two most frequently dispensed medicines in the class among adults with ADHD. RASSi = renin-angiotensin-aldosterone system inhibitor.

In 2021, 16.5% of adults with ADHD were dispensed at least one cardiovascular medicine. The prevalence of cardiovascular medicine use among people with ADHD increased with advancing age; it ranged from 6.0% among people aged 18 to 29 years, to 15.1% aged 30 to 49 years, then 42.5% aged 50 to 64 years, rising to 72.6% aged ≥65 years. Compared to those without ADHD, adults with ADHD had a higher prevalence of cardiovascular medicine use, yielding an aPR of 1.7 (16.5% vs. 10.0%, 95% CI [1.6, 1.7]). This difference in use was evident for both females (18.9% vs. 11.1%, aPR = 1.7; 95% CI [1.6, 1.8]) and males (15.0% vs. 9.3, aPR = 1.6; 95% CI [1.5, 1.7]; Figure 1). While the prevalence of cardiovascular medicine use increased with advancing age regardless of ADHD status, the relative difference in prevalence was most pronounced among people aged 18 to 29 years (6.0% vs. 2.2%, aPR = 2.8, 95% CI [2.4, 3.1]), decreasing with each successive age groups to a modest difference among people aged ≥65 (72.6% vs. 59.2%; aPR = 1.2, 95% CI [1.1, 1.3]). Females with ADHD had slightly higher prevalence than males with ADHD (18.9% vs. 15.0%; Figure 1). Antihypertensives (e.g., RASSi, calcium-channel blockers, and beta-blockers) and lipid modifiers were the most common cardiovascular medicines regardless of ADHD status (Figure 2). Comparing females with ADHD to males with ADHD, we observed a higher prevalence for a few specific medicines: propranolol (4.4% vs. 2.5%), furosemide (1.4% vs. 0.5%), and spironolactone (1.4% vs. 0.5%; Table 2).

**Table 2.** Top 20 Most Used Cardiovascular Medicines Among Australian Adults With ADHD, by Sex.

Female (*N* = 5,571)					Male (*N* = 9182)				
	Common CVD medicine	Medicine type	*n*	Prevalence (%)		Common CVD medicine	Medicine type	*n*	Prevalence (%)
1	Propranolol	Nonselective beta-blocker	246	4.4	1	Rosuvastatin	Lipid modifier	301	3.3
2	Rosuvastatin	Lipid modifier	190	3.4	2	Perindopril	RASSi (ACEI)	243	2.6
3	Perindopril	RASSi (ACEI)	126	2.3	3	Amlodipine	Calcium-channel blocker	233	2.5
4	Atorvastatin	Lipid modifier	126	2.3	4	Propranolol	Nonselective beta-blocker	233	2.5
5	Amlodipine	Calcium-channel blocker	97	1.7	5	Atorvastatin	Lipid modifier	222	2.4
6	Furosemide	Loop diuretic diuretics	77	1.4	6	Telmisartan	RASSi	115	1.3
7	Spironolactone	Potassium-sparing diuretic	76	1.4	7	Hydrochlorothiazide	Low-ceiling diuretic	112	1.2
8	Metoprolol	Selective beta-blocker	74	1.3	8	Ramipril	RASSi (ACEI)	99	1.1
9	Candesartan	RASSi (ARB)	71	1.3	9	Metoprolol	Selective beta-blocker	99	1.1
10	Telmisartan	RASSi (ARB)	70	1.3	10	Candesartan	RASSi (ARB)	88	1.0
11	Hydrochlorothiazide	Low-ceiling diuretic	67	1.2	11	Irbesartan	RASSi (ARB)	78	0.8
12	Enoxaparin	Antithrombotic agent	66	1.2	12	Atenolol	Selective beta-blocker	70	0.8
13	Irbesartan	RASSI (ARB)	57	1.0	13	Olmesartan	RASSi	60	0.7
14	Atenolol	Selective beta-blocker	50	0.9	14	Ezetimibe	Lipid modifier	60	0.7
15	Ezetimibe	Lipid modifier	36	0.6	15	Fenofibrate	Lipid modifier	54	0.6
16	Rivaroxaban	Antithrombotic agent	36	0.6	16	Valsartan	RASSi (ARB)	47	0.5
17	Ramipril	RASSi (ACEI)	31	0.6	17	Furosemide	Loop diuretic	46	0.5
18	Olmesartan	RASSi (ARB)	29	0.5	18	Clopidogrel	Antithrombotic agent	45	0.5
19	Apixaban	Antithrombotic agent	25	0.4	19	Spironolactone	Potassium-sparing diuretic	43	0.5
20	Lercanidipine	Calcium blocker	23	0.4	20	Enoxaparin	Antithrombotic agent	42	0.5

*Note*. RASSi = renin-angiotensin-aldosterone system inhibitor; ACEI = angiotensin-converting enzyme, inhibitor; ARB = angiotensin receptor blocker.

### Prevalence of Cardiovascular Medicine Use by Type

**Figure 3.**
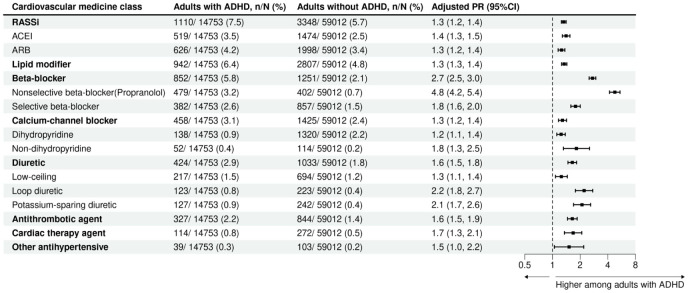
Prevalence of cardiovascular medicine use according to type, among Australian adults with and without ADHD, matched on age and sex. *Note*. Estimates from a 10% sample of people eligible for subsidised medicines. For loop diuretics, furosemide was the only subtype identified in the observed data. PRs were adjusted for age and sex. ATC = anatomical therapeutic chemical; PR = prevalence ratio; RASSi = renin-angiotensin-aldosterone system inhibitor; ACEI = angiotensin-converting enzyme inhibitor; ARB = angiotensin receptor blocker.

Adults with ADHD had a higher prevalence of use across all cardiovascular medicine types than those without ADHD (Figure 3). Certain medicines were markedly more prevalent among adults with ADHD than those without ADHD, for example, beta-blockers (5.8% vs. 2.1%; aPR = 2.7, 95% CI [2.5, 3.0]), and, in particular, the nonselective beta-blocker, propranolol (3.2% vs. 0.7%; aPR = 4.8, 95% CI [4.2, 5.4]; Supplemental eTables S4 and S5).

Additionally, we observed elevated relative differences in the use of loop diuretics (0.8% vs. 0.4%; aPR = 2.2, 95% CI [1.8, 2.7]), potassium-sparing diuretics (0.9% vs. 0.4%; aPR = 2.1, 95% CI [1.7, 2.6]), selective beta-blockers (2.6% vs. 1.5%; aPR = 1.8, 95% CI [1.6, 2.0]), non-dihydropyridine calcium-channel blockers (0.4% vs. 0.2%; aPR = 1.8, 95% CI [1.3, 2.5]), cardiac therapy agents (0.8% vs. 0.5%; aPR = 1.7, 95% CI [1.3, 2.1]), and antithrombotic agents (2.2% vs. 1.4%; aPR = 1.6, 95% CI [1.5, 1.9]; Figure 3). Among these medicines, the elevated use of loop diuretics and potassium-sparing diuretics was particularly evident among females with ADHD (loop diuretics: 1.4% vs. 0.5%; aPR = 2.8, 95% CI [2.1, 3.7]; potassium-sparing diuretics: 1.5% vs. 0.6%; aPR = 2.5, 95% CI [1.9, 3.3]) whilst also elevated in males with ADHD (0.5% vs. 0.3%; aPR = 1.6, 95% CI [1.2, 2.3]; 0.5% vs. 0.3%; aPR = 1.6, 95% CI [1.1, 2.3], respectively). Propranolol use was strongly associated with ADHD in both females and males (females 4.4% vs. 1.0%; aPR = 4.4, 95% CI [3.7, 5.2]; males 2.5% vs. 0.5%; aPR = 5.3, 95% CI [4.3, 6.4]; Supplemental eFigure S3).

### Post Hoc Analysis

We conducted a post hoc analysis excluding propranolol, given it is commonly used off-label for non-cardiovascular conditions, including psychiatric symptoms (Boyce et al., 2021; Lin et al., 2006). In this analysis, ADHD remained consistently associated with increased cardiovascular medicine use overall (13.9% vs. 9.5%, aPR = 1.5, 95% CI [1.4, 1.5]), with similar patterns by sex and age. Although we observed a decrease in the magnitude of the association relative to the main analysis in females (15.4% vs. 10.3%, aPR = 1.5, 95% CI [1.4, 1.6]) and younger adults aged 18 to 29 years (3.6% vs. 1.6%, aPR = 2.2, 95% CI [1.9, 2.6]) and 30 to 49 years (12.0% vs. 7.6%, aPR = 1.6, 95% CI [1.5, 1.7]), the association remained strongest among young adults aged 18 to 29 years (Supplemental eFigure S4).

## Discussion

In this nationwide population-based study from Australia, the prevalence of cardiovascular medicine use among adults with ADHD was 1.7-fold higher than those without ADHD. This elevated use of cardiovascular medicines in ADHD was clear for people across all sex- and age groups, and persisted in post hoc analyses excluding propranolol, commonly prescribed off-label for non-cardiovascular conditions. Further, the stark 2.8-fold prevalence we observed in cardiovascular medicine use among young adults with and without ADHD warrants detailed investigation. In terms of specific medicines, use of antithrombotic agents, cardiac therapy agents and specific diuretics (i.e., loop diuretics [furosemide] and potassium-sparing diuretics) was noticeably increased in adults with ADHD, with this pattern being particularly evident in females with ADHD.

Our main findings of cardiovascular medicine use in 2021 were consistent with a cross-sectional study from Sweden, showing an overall higher medicine use during 2013 among adults with ADHD compared to those without ADHD (18.2% vs. 5.9%; Zhang et al., 2021). Similar to our findings, they also reported a pronounced prevalence difference in younger age groups, with the association attenuating with age.

Four possible reasons for elevated cardiovascular risk among people with ADHD have been described in the literature: (1) The core symptoms of ADHD, such as impulsivity and inattention, are linked to unhealthy behaviours and poor dietary habits (Li et al., 2022). (2) A potential genetic link between ADHD and cardiovascular conditions may, in part, explain the increased risk, as suggested by recent genome-wide association studies (Du Rietz et al., 2021; Garcia-Argibay et al., 2022). (3) Psychiatric conditions comorbid with ADHD are associated with cardiovascular risks, through physiological pathways and psychotropic medicine use (Penninx & Lange, 2018). Indeed, we observed an increased proportion of psychotropic use and psychiatric comorbidities at baseline. (4) ADHD medicines’ impact on the cardiovascular system, evidence for which remains inconsistent. Meta-analyses of randomised clinical trials have shown short-term rises in heart rate and blood pressure with both psychostimulants and non-psychostimulant ADHD medicines (Zhang et al., 2022, 2024). Recent meta-analyses of observational studies, with relatively short follow-up periods, have found non-significant increases in cardiac arrest and heightened heart rate (Zhang et al., 2022). On the other hand, the latest long-term longitudinal study identified a 4% yearly dose-response increase in CVD with ADHD medicine use (Zhang et al., 2024).

In sum, the accumulating evidence of a clear link of ADHD – and treatment thereof – with cardiovascular conditions, underscores the importance of regular cardiovascular assessments of crucial parameters such as blood pressure and lipid profiles in people diagnosed with ADHD. Our findings highlight the importance of effective cardiovascular screening within this population, particularly in young adults.

Our analysis identified an elevated use of cardiovascular medicine among young adults with ADHD, indicating a potential vulnerability in this demographic. Notably, propranolol exhibited a markedly higher prevalence among younger adults with ADHD compared to those without. This nonselective beta-blocker is often used for anxiety and other non-cardiovascular conditions, with a secondary data analysis from the US suggesting approximately 60% of its prescriptions for off-label uses (Lin et al., 2006). Even though the elevated use of cardiovascular medicine in this age group was partly driven by non-cardiovascular use of propranolol, the trends of heightened cardiovascular medicine use among adults with ADHD remained after excluding this medicine. Explanations for age-specific challenges for young adults with ADHD may be associated with risk-taking behaviours common in younger individuals with ADHD (Agnew-Blais et al., 2018; Treur et al., 2021; Young et al., 2020). Substance use, poor mental health, and productive events could serve as cardiovascular risks (Agnew-Blais et al., 2018; Choi et al., 2022; Young et al., 2020). More frequent contact with the healthcare system, compared to counterparts without ADHD, could also play a role in earlier detection and treatment of cardiometabolic conditions in the younger age groups.

Our analysis uncovered distinctive patterns in the use of specific types and subtypes of cardiovascular medicines among adults with ADHD. Interestingly, we observed elevated PRs for antithrombotic agents, loop diuretics (furosemide), potassium-sparing diuretics, non-dihydropyridine calcium-channel blockers, and cardiac therapy agents, medicines often indicated for secondary prevention (NHFA CSANZ Heart Failure Guidelines Working Group et al., 2018). In contrast, first-line cardiovascular medicines, including lipid modifiers and antihypertensives, such as RASSi, calcium-channel blockers and low-ceiling diuretics (National Heart Foundation of Australia, 2016) had modest PRs. These results may indicate more severe cardiovascular conditions (e.g., heart failure and ischaemic heart disease) among people with ADHD, but should be interpreted with caution as we observed low prevalence of use for most subclasses with elevated PRs (<1.0%, except for antithrombotic) and that furosemide (loop-diuretics) and spironolactone (potassium-sparing diuretics) could be used for non-cardiovascular conditions (Butt et al., 2022; Ganson et al., 2023).

Our study findings revealed sex-specific variations in medicine use, notably with females with ADHD showing a higher prevalence of propranolol (nonselective beta-blocker), furosemide (loop diuretics), and spironolactone (potassium-sparing diuretics) than their male counterparts. These differences may relate to sex-specific risk profiles, such as biological, hormonal, and symptomatic features of ADHD and CVD (Okoth et al., 2020; Vogel et al., 2021; Young et al., 2020), as well as behavioural differences such as using furosemide and spironolactone for aesthetic reasons (O’Dea & Cinelli, 2016).

### Strengths and Limitations

This study draws strength from a nationally representative sample of Australians and their dispensing claims that comprehensively capture ADHD and cardiovascular medicine use. Nevertheless, it has limitations particularly associated with the study design and the data source. Firstly, given the descriptive nature of our analyses and the cross-sectional design, our findings do not examine the temporal relationship between ADHD treatment and cardiovascular medicine use, nor do we imply causality. Future research with longitudinal data and detailed medicine histories is necessary, including investigating the possibility that concomitant psychotropic medicine use may mediate increased cardiovascular medicine use. Furthermore, we acknowledge that the profile of medicine use may vary by other factors we were unable to measure, such as CVD diagnoses, socioeconomic status, health behaviours and frequency of healthcare contact, which may be associated with the observed increased CVD medicine use. Secondly, we acknowledge limitations resulting from misclassification in the cohort due to the absence of a recorded ADHD diagnosis. The study cohort was identified based on a history of pharmacological treatment for the condition using dispensing data, overlooking those who were undiagnosed, untreated, treated non-pharmacologically, or did not meet the requirements for medicine subsidies. Nevertheless, any contamination by individuals with ADHD assigned to the comparison group is likely mitigated by the much larger number of individuals without ADHD, thus minimising its impacts on the overall results. Thirdly, the absence of information on cardiovascular medicine indications may introduce misclassification bias. This is relevant given the potential for off-label use, particularly for medicines such as propranolol, which is widely used for psychiatric indications and may also be prescribed to mitigate adverse effects of stimulants, such as increased heart rate and anxiety; hence, we addressed this through our post hoc analysis. This may also be the case for some diuretics; for instance, spironolactone may be used off-label for dermatological conditions (Butt et al., 2022). In addition, furosemide might be misused for weight loss among young females instead of the treatment of CVD (Ganson et al., 2023). Nonetheless, unlike propranolol, the prevalence of these diuretics was low, and any use for non-cardiovascular conditions was unlikely to impact the overall results. Despite these limitations, we believe the findings will generalise to other jurisdictions with similar healthcare systems and clinical practice.

## Conclusion

This nationwide cross-sectional study identified elevated cardiovascular medicine use in adults with ADHD, especially in younger people. Our findings point to a high use of cardiovascular medicines among people with ADHD and highlight the importance of regular monitoring and management of cardiovascular health in this population throughout the lifespan.

## Supplemental Material

sj-docx-1-jad-10.1177_10870547261418763 – Supplemental material for Cardiovascular Medicine Use Among Adults With ADHD: A Nationwide Study in Australia Supplemental material, sj-docx-1-jad-10.1177_10870547261418763 for Cardiovascular Medicine Use Among Adults With ADHD: A Nationwide Study in Australia by Masako Araki, Helga Zoega, Malcolm Gillies, Michael O. Falster, David Peiris, Sallie-Anne Pearson, Henrik Larsson and Juliana de Oliveira Costa in Journal of Attention Disorders

sj-jpg-2-jad-10.1177_10870547261418763 – Supplemental material for Cardiovascular Medicine Use Among Adults With ADHD: A Nationwide Study in Australia Supplemental material, sj-jpg-2-jad-10.1177_10870547261418763 for Cardiovascular Medicine Use Among Adults With ADHD: A Nationwide Study in Australia by Masako Araki, Helga Zoega, Malcolm Gillies, Michael O. Falster, David Peiris, Sallie-Anne Pearson, Henrik Larsson and Juliana de Oliveira Costa in Journal of Attention Disorders

sj-jpg-3-jad-10.1177_10870547261418763 – Supplemental material for Cardiovascular Medicine Use Among Adults With ADHD: A Nationwide Study in Australia Supplemental material, sj-jpg-3-jad-10.1177_10870547261418763 for Cardiovascular Medicine Use Among Adults With ADHD: A Nationwide Study in Australia by Masako Araki, Helga Zoega, Malcolm Gillies, Michael O. Falster, David Peiris, Sallie-Anne Pearson, Henrik Larsson and Juliana de Oliveira Costa in Journal of Attention Disorders

sj-jpg-4-jad-10.1177_10870547261418763 – Supplemental material for Cardiovascular Medicine Use Among Adults With ADHD: A Nationwide Study in Australia Supplemental material, sj-jpg-4-jad-10.1177_10870547261418763 for Cardiovascular Medicine Use Among Adults With ADHD: A Nationwide Study in Australia by Masako Araki, Helga Zoega, Malcolm Gillies, Michael O. Falster, David Peiris, Sallie-Anne Pearson, Henrik Larsson and Juliana de Oliveira Costa in Journal of Attention Disorders

sj-jpg-5-jad-10.1177_10870547261418763 – Supplemental material for Cardiovascular Medicine Use Among Adults With ADHD: A Nationwide Study in Australia Supplemental material, sj-jpg-5-jad-10.1177_10870547261418763 for Cardiovascular Medicine Use Among Adults With ADHD: A Nationwide Study in Australia by Masako Araki, Helga Zoega, Malcolm Gillies, Michael O. Falster, David Peiris, Sallie-Anne Pearson, Henrik Larsson and Juliana de Oliveira Costa in Journal of Attention Disorders
