# Supplementary figures and images for: Cardiovascular Medicine Use Among Adults With ADHD: A Nationwide Study in Australia
Source: J Atten Disord. 2026 Mar 27;30(6):773–83. doi: 10.1177/10870547261418763 (PMC13110325; doi:10.1177/10870547261418763)

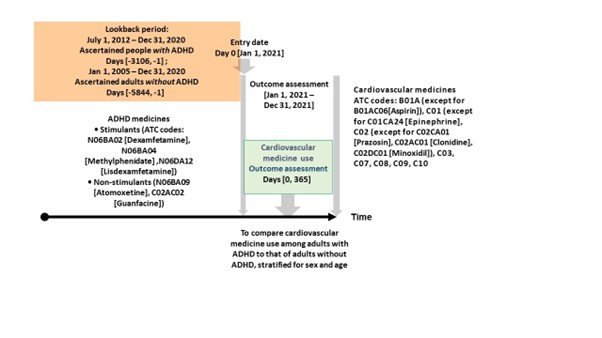

Supplement: sj-jpg-2-jad-10.1177_10870547261418763 – Supplemental material for Cardiovascular Medicine Use Among Adults With ADHD: A Nationwide Study in Australia [file sj-jpg-2-jad-10.1177_10870547261418763.jpg]

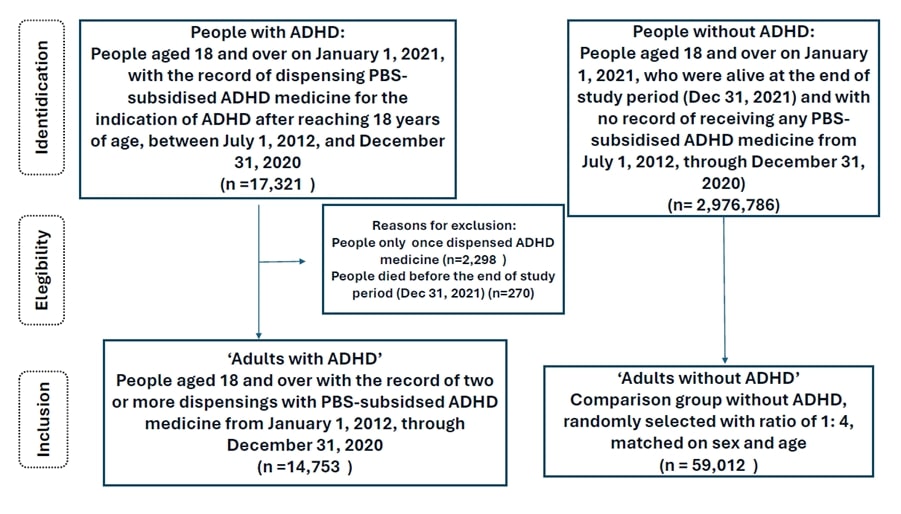

Supplement: sj-jpg-3-jad-10.1177_10870547261418763 – Supplemental material for Cardiovascular Medicine Use Among Adults With ADHD: A Nationwide Study in Australia [file sj-jpg-3-jad-10.1177_10870547261418763.jpg]

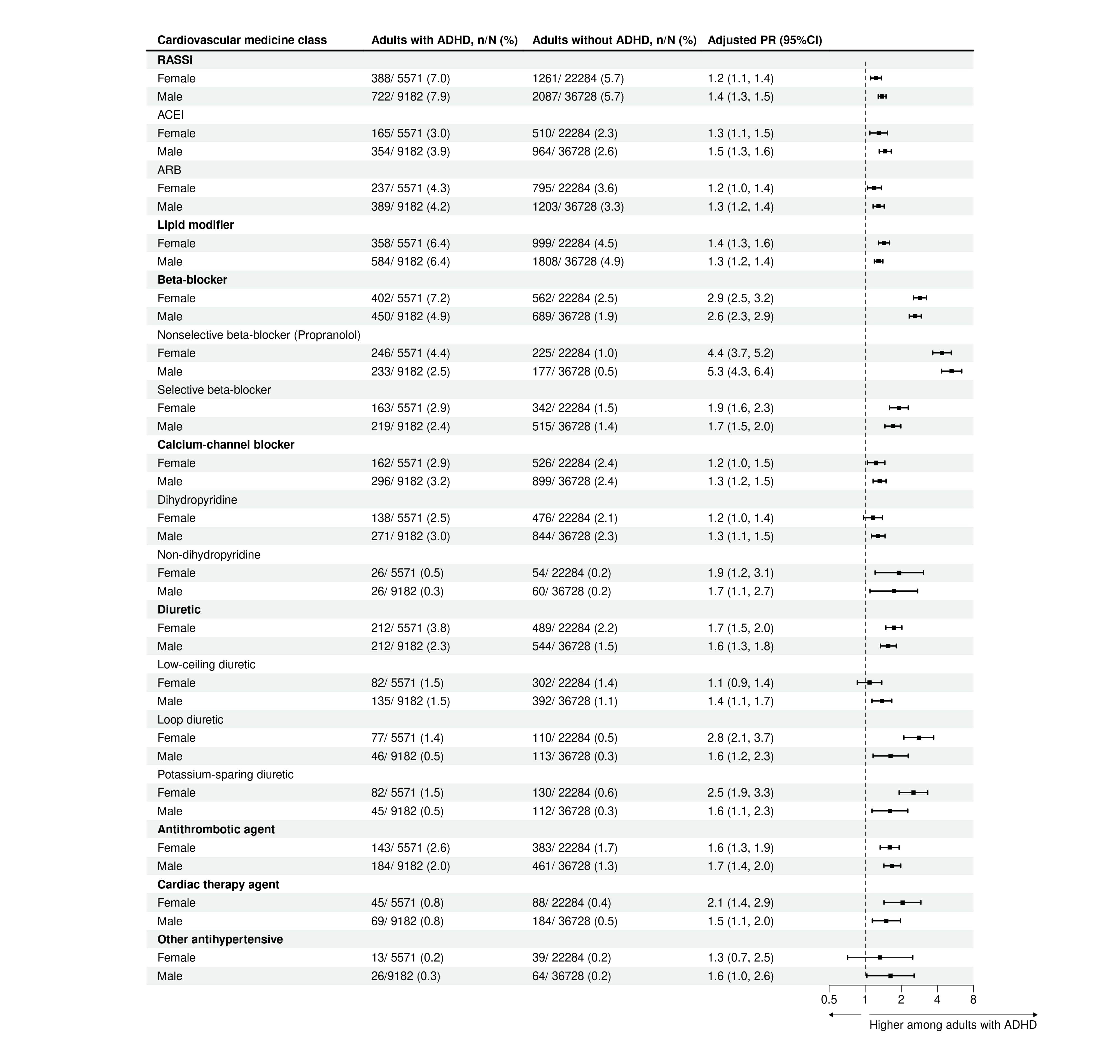

Supplement: sj-jpg-4-jad-10.1177_10870547261418763 – Supplemental material for Cardiovascular Medicine Use Among Adults With ADHD: A Nationwide Study in Australia [file sj-jpg-4-jad-10.1177_10870547261418763.jpg]

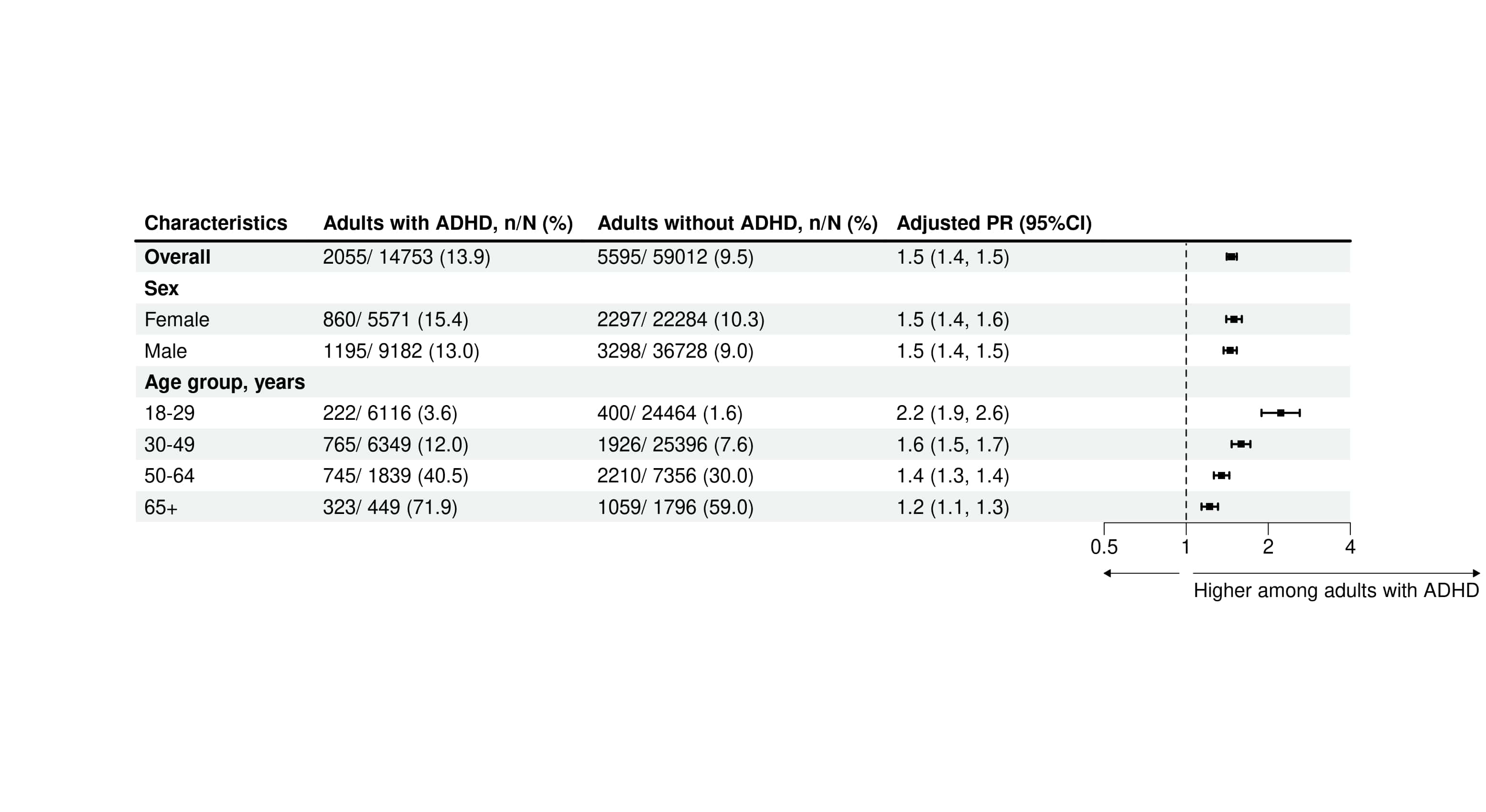

Supplement: sj-jpg-5-jad-10.1177_10870547261418763 – Supplemental material for Cardiovascular Medicine Use Among Adults With ADHD: A Nationwide Study in Australia [file sj-jpg-5-jad-10.1177_10870547261418763.jpg]
